# Healthcare access satisfaction before and during the COVID-19 pandemic among Peruvian children with down syndrome

**DOI:** 10.1186/s12887-025-05990-1

**Published:** 2025-10-28

**Authors:** Tania Vasquez-Loarte, Gabriela A. Guerra, Enrique M. Saldarriaga, Lucero Torres-Gomez, Elizabeth J. Ramos-Orosco, Emiliana Rizo-Patrón, Gioconda Manassero-Morales, German F. Alvarado

**Affiliations:** 1 Department of Pediatrics, Wyckoff Heights Medical Center, Brooklyn, NY USA; 2 Facultad de Medicina Humana, Universidad Peruana Cayetano Heredia, Lima, Perú; 3 The Comparative Health Outcomes, Policy, and Economics (CHOICE) Institute, University of Washington, Seattle, WA USA; 4 Facultad de Medicina, Universidad Peruana de Ciencias Aplicadas, Santiago de Surco, Lima Perú; 5 Department of Pediatrics, Nationwide Children’s Hospital, The Ohio State University College of Medicine, Columbus, OH USA; 6 Subunidad de Investigación e Innovación Tecnológica, Instituto Nacional de Salud del Niño-San Borja, San Borja, Lima Perú; 7 Departamento de Genética, Instituto Nacional de Salud del Niño-San Borja, San Borja, Lima Perú; 8 Facultad de Salud Pública y Administración “Carlos Vidal Layseca”, Universidad Peruana Cayetano Heredia, Lima, Perú

**Keywords:** Down Syndrome, COVID-19, Global health, Healthcare access, Trisomy 21, Health services

## Abstract

**Background:**

In Peru, more than 9,000 individuals with Down syndrome (DS) experienced disruptions in healthcare access due to strict limitations on appointment availability and widespread hospital and clinic closures during the COVID-19 pandemic. This study examines changes in parental satisfaction with healthcare access before and during the pandemic (2021 vs. 2019), as reported by caregivers of children with DS.

**Methods:**

We conducted a cross-sectional survey between October 2022 and April 2023 among parents of children with DS, aged 2–12 years, affiliated with the Peruvian Association for Children with Down Syndrome. The survey collected information on family demographics, comorbidities, medical care, and satisfaction with healthcare access. The primary outcome was the change in parental satisfaction between 2019 and 2021. The secondary outcome assessed healthcare utilization. Linear regression analysis was used to evaluate associations between healthcare utilization and satisfaction, adjusting for type of disability, parental education level, and type of health insurance.

**Results:**

A total of 223 participants were included in the analysis. Developmental delay (75%) and cardiac conditions (41%) were the most commonly reported comorbidities. In 2019, 68.2% of children (*n* = 152) attended at least one medical appointment, compared to 53.8% (*n* = 120) in 2021. Among children with visits in both years (*n* = 72), satisfaction with healthcare access declined by 10.9 points (95% CI: 4.8–16.9; *p* < 0.01). For those receiving pediatric care during both years (*n* = 43), satisfaction decreased by 11.8 points (95% CI: 3.4–20.1; *p* < 0.01). In 2021, all specialties except pediatrics showed a discrepancy between needed and received care, with early intervention services showing the largest gap.

**Conclusions:**

The COVID-19 emergency significantly impacted healthcare access satisfaction among children with DS. Strengthening pediatric training in genetic conditions is critical, particularly in low-resource settings where access to specialists is limited.

**Supplementary Information:**

The online version contains supplementary material available at 10.1186/s12887-025-05990-1.

## Introduction

The COVID-19 pandemic negatively impacted healthcare access globally, with low- and middle-income countries (LMIC) experiencing disruptions due to preexisting limitations in infrastructure and resources [1]. Healthcare utilization declined by 30%, leading to a 50% reduction in medical appointments and treatments. Children under 10 years old experienced a 72% decline in preventive and curative medical services [2], which contributed to a 9.8–44.7% increase in under-five mortality rate in LMIC [3, 4].

Down syndrome is a genetic condition that affects 1 per 1,000 live births worldwide (UN 2022). In Peru, by 2020, 17,913 people with Down Syndrome were registered in the National Committee for People with Disabilities (CONADIS) [5]. Over the past two decades, the Peruvian Association for Children with Down Syndrome and CONADIS have developed healthcare policies and advocacy efforts on behalf of this population [6]. Down syndrome is associated with well-documented comorbidities, for which evidence-based clinical protocols have been established. The Peruvian Guideline for the Management of Children with Down Syndrome aligns with the AAP recommendations that consist of biannual health maintenance, and annual ophthalmology, dental, hearing, hemoglobin and thyroid hormone evaluations [7, 8]. However, the implementation of these guidelines remains a challenge in LMIC, even outside of public health emergency conditions. Furthermore, the COVID-19 pandemic exacerbated healthcare disparities, as individuals with Down Syndrome were more susceptible to moderate to severe COVID-19 illness, increasing their overall healthcare needs [9–11].

In Peru, substantial healthcare changes during 2020 and 2021 prioritized acute medical care for COVID-19 patients, limiting health maintenance visits for the pediatric population, including children with Down Syndrome [12–14]. Understanding the changes on healthcare access and satisfaction for this specific population is critical for informing patient-centered care and strengthening health system preparedness in future public health emergencies. Moreover, given the overlap in healthcare needs between children with Down syndrome and those with other genetic conditions, insights from this study may help identify broader barriers to continuity of care in LMICs.

This study aims to assess changes in parental satisfaction with healthcare access for children with Down syndrome, comparing data from 2019 to 2021.

## Methods

Parents of children with Down Syndrome, aged 2 to 12 years old in 2019, and affiliated with the Peruvian Association for Children with Down Syndrome, were recruited as survey participants. This organization was selected due to its role as a central registry for children with Down Syndrome in Peru and its longstanding reputation of ethical conduct, which instilled confidence in the potential participants. The association provided a list of members born between 2007 and 2017. Only the principal investigator and lead author had access to the full list of participants.

### Study sample

The dataset provided by the Association comprised 631 eligible individuals, who were stratified by age and 315 were randomly contacted. Each parent was called up to three times. Upon consent, parents chose to complete the survey either online or by phone. Parents who could not be reached after three attempts or who declined to participate were excluded. Of these, 83 respondents met exclusion criteria and 9 submitted incomplete surveys. The final sample comprised 223 participants. This sample size provided 94% power to detect a 10-point difference in the paired mean satisfaction score, assuming a 95% confidence level (Supplementary Figure).

### Survey

The survey contained 41 questions covering demographics, medical history, healthcare access (healthcare utilization, medical care gap, and barriers), and perceived satisfaction. Survey development was informed by clinical guidelines for children and adolescents with Down Syndrome (Supplementary data 1) [7, 8]. Medical conditions were self-reported by parents to facilitate recall and reflect their familiarity with their children’s health status. Intellectual disability diagnosis was confirmed through the CONADIS registration card. Early intervention services refers to occupational and physical therapies. In the section about barriers, “prior authorization” referred to insurance approvals for diagnostic procedures and medications. Barrier categories were adapted from Imbachi et al. 2020 [15]. We hypothesized that perceived satisfaction and healthcare access would differ in 2021 compared to 2019 due to the effects of the COVID-19 pandemic.

### Analysis

The primary outcome was the change in healthcare access satisfaction between 2019 and 2021. Satisfaction was measured using the question: “How satisfied were you with the healthcare access you experienced?” rated on a scale from 1 (least satisfied) to 10 (most satisfied) [16–18]. The secondary outcome was healthcare utilization, defined as the percentage of children who had at least one medical appointment, specifically within pediatric care. We also evaluated the gap in medical care, defined as the unmet need for medical services. This was calculated using the formula:

$$\left[1\;-\;\left(children\;who\;accessed\;care/children\;who\;needed\;care\right)\right]\;\times\;100$$

Descriptive statistics included frequencies, percentages, means, and standard deviations. To assess differences between 2019 and 2021, we used paired *t*-tests for continuous variables and Chi-square or McNemar tests for categorical variables. Linear regression was applied, to determine which covariates predicted patient satisfaction. Standard errors in the linear regressions were clustered by respondents to account for the lack of independence in the observations arising from the same person in two points of time [19]. For data analysis, we used IBM SPSS Statistics (Version 27) [20] and R4.3.3 [21].

### Ethics

The study protocol and informed consent process were approved by the Ethics Committee at Universidad Peruana Cayetano Heredia (IRB No. 206405). During recruitment, a verbal explanation of the study was provided by phone, and verbal consent was obtained before administering the survey. No personal identifying information, images, or videos were collected, and consent for publication was not required. Participation did not affect the families’ relationship with the Association, and no financial incentives were provided.

## Results

### Children with down syndrome and parental demographics

A total of 223 parents of children with Down Syndrome participated in the survey from October 2022 to April 2023. Of the children, 42% were female (94/223) and 57% (128/223) were male. The median age was 4 years (IQR 3–7) in 2019 and 6 years (IQR 5–9) in 2021.

95% (212/223) had received some form of education: preschool (107/212, 50.0%), elementary school (95/212, 44.8%), and secondary school (10/212, 4.7%). 70% (157/223) attended specialized education centers. The majority (154/223, 69%) were born in the capital city, while 31% (69/223) were born in other Peruvian cities.

The most frequently reported conditions were speech delay (163/223, 73.1%), congenital heart disease (93/223, 41.8%), and motor delay (90/223, 40%). Most children (182/223, 81%) were registered with the National Council for Persons with Disabilities. The reported degree of disability was mild in 25.1%, moderate in 34.5% (*n* = 77), severe in 24.2% (*n* = 54), and unknown in 16.1% (*n* = 36).

The median caregiver age was 42.9 years (± 8.6), mostly female (189/223, 84.7%), and biological parents (222/223, 99.6%). Educational attainment included high school (70/223, 31.3%), associate’s degree (57/223, 25.6%), and university education (94/223, 42.2%). The most commonly used sources of medical information were the internet (141/223, 63.2%), parent groups (139/223, 62.3%), and healthcare providers (120/223, 53.8%).

### Healthcare utilization

In 2019, 68% (152/223) of children had at least one medical appointment, decreasing to 53.8% (120/223) in 2021. A total of 43% (96/223) had appointments in both years. There were no significant differences in appointment rates between those living in Lima versus other cities in 2019 (69%, 107/155, 65%, 45/68, p 0.3).

Pediatric care appointments were reported by 46% (103/223) in 2019 and 39.5% (88/223) in 2021 (*p* = 0.1); 25.5% (57/223) had appointments in both years. In 2019, children with cardiac or ENT conditions were more likely to have a medical visit than those without (46.7% vs. 31%, *p* = 0.02; 45.4% vs. 31%, *p* = 0.04, respectively). Most 2019 appointments were in-person (90.8%) rather than via telemedicine (9.2%, *p* < 0.01).

In 2021, having hypothyroidism or identifying the medical provider as the main source of health information was associated with higher appointment rates (10.7% vs. 21.7%, p 0.02; and 42.7% vs. 63.3%, *p* < 0.01). Use of telemedicine significantly increased in 2021 (61.6% vs. 35.9%, *p* < 0.01).

Overall, the characteristics of children who had at least one appointment were similar across years, with exceptions for telemedicine use (2021: 61.6% vs. 2019: 9.2%, *p* < 0.01) and provider information reliance (63.3% vs. 57.2%, *p* = 0.01) (Table 1).

**Table 1:**
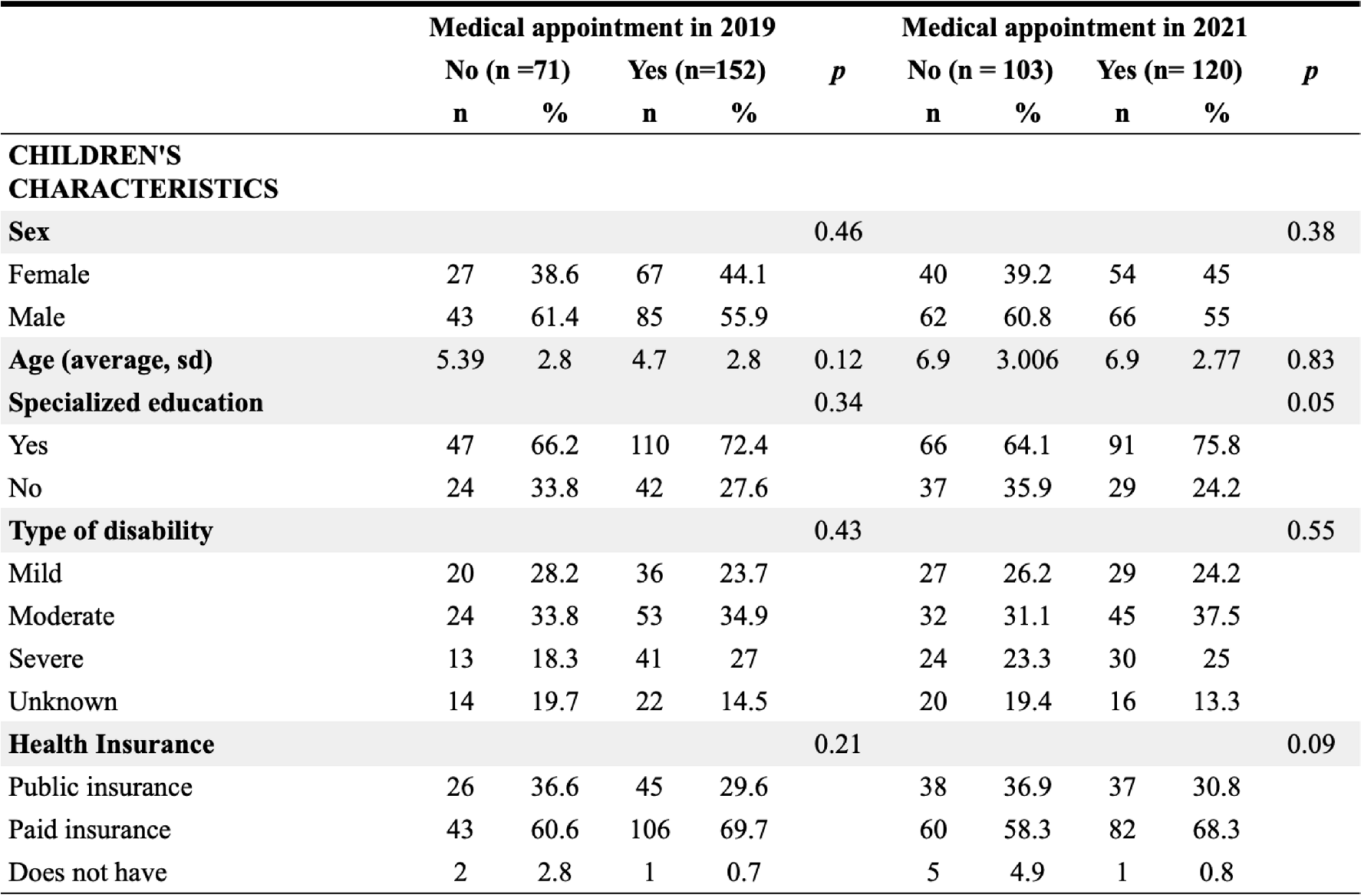
Characteristics of children with down syndrome and their parents

Note: 222 participants gave information about the child’s gender

### Healthcare satisfaction scores

Satisfaction data were completed by 73.1% of participants (*n* = 162). Mean satisfaction scores were significantly higher in 2019 than in 2021 (55.7 ± 27.0 vs. 46.4 ± 28.9, *p* < 0.001). This difference remained when analyzing the entire sample (*n* = 223). Participants whose children had a pediatric appointment reported higher satisfaction scores than those who attended other specialties (2019: 61.3 ± 25.4 vs. 55.8 ± 27.1; 2021: 48.7 ± 27.3 vs. 46.4 ± 28.9).

In 2019, families residing in the capital city reported higher satisfaction scores than those from other regions (61.1 ± 25.3 vs. 53.0 ± 28.1, *p* = 0.04). No such difference was found for 2021. Satisfaction did not differ significantly by type of insurance, education level, child’s age, or disability category.

### Gaps in medical care access

Unmet medical needs were significantly higher in 2021 than in 2019 (18.4% vs. 4.2%, *p* < 0.01). Gaps increased across all specialties except pediatrics. In 2019, cardiology (36%) and ophthalmology (21%) showed the highest gaps, while pediatrics (9%) and early intervention (8%) showed the lowest. In 2021, cardiology (57%), hematology (42%), and speech therapy (36%) had the highest gaps, with no gap reported in pediatrics (Graph 1).

Among children with congenital heart disease (*n* = 93), cardiology appointment access declined from 37.6% (*n* = 35) in 2019 to 11.8% (*n* = 11) in 2021 (*p* < 0.001).

### Healthcare utilization and patient satisfaction by year

Bivariate analysis showed that having a medical appointment in 2021 was associated with an average 10-point decrease in satisfaction compared to 2019 (− 10.77 ± 2.23, 95% CI − 15.14 to − 6.4). The reduction persisted among subgroups with appointments in any specialty (*n* = 72, − 11 ± 2.97) and in pediatrics (*n* = 43, − 12.5 ± 3.81).

Multivariate analyses adjusted for disability severity, parental education, and insurance type confirmed these findings (Table 2).

**Table 2:**
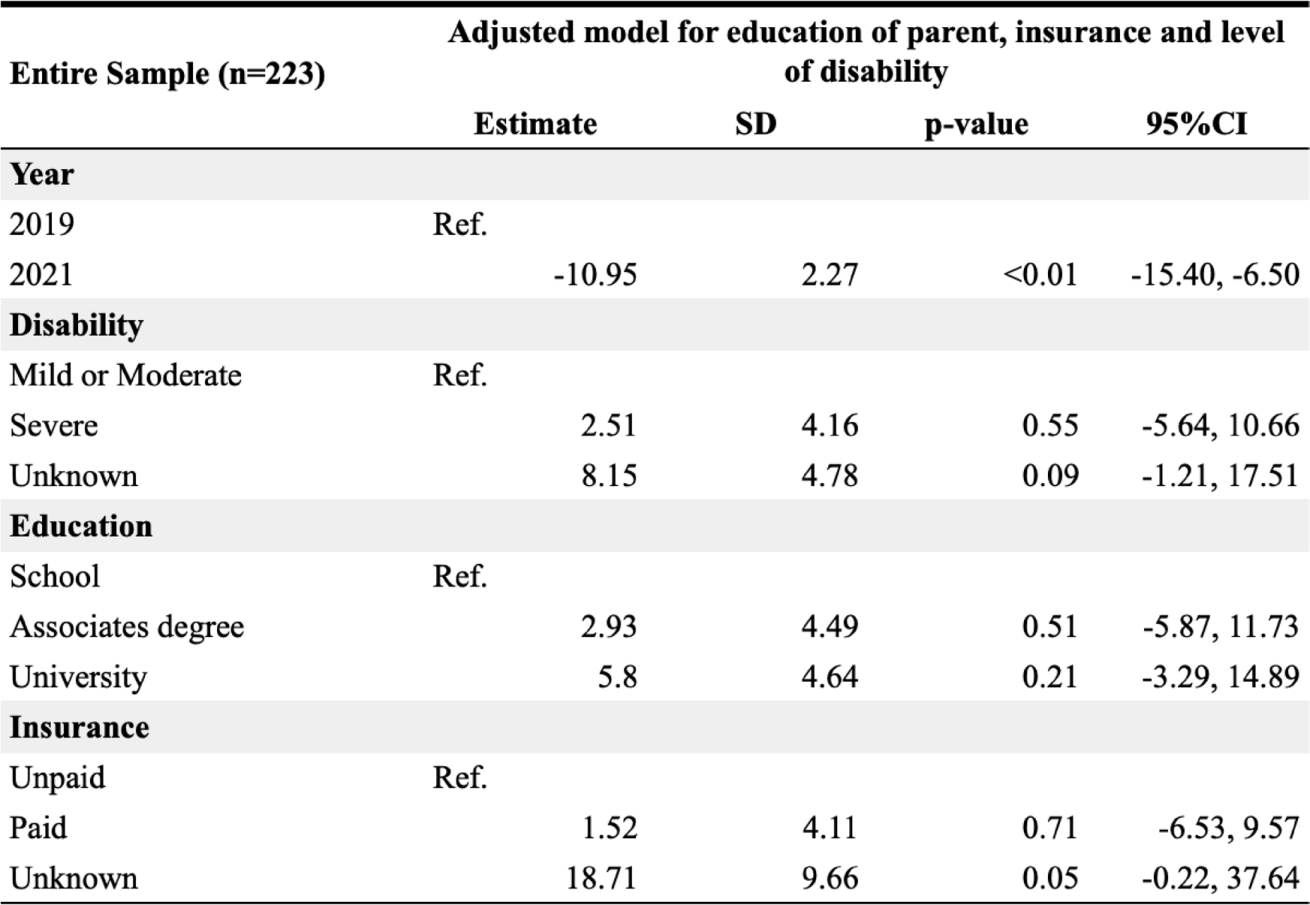
Association between perceived satisfaction, year of medical care and demographic covariates

Telemedicine use in 2021 was associated with slightly higher satisfaction scores than in-person care, although the sample size was small (*n* = 11 in 2019 vs. *n* = 60 in 2021).

### Barriers towards access to medical care

The most frequently reported barriers in 2021 compared to 2019 included referral difficulties (*n* = 35, 48.6% vs. *n* = 28, 40.2%), transportation issues (*n* = 21, 30.5% vs. *n* = 16, 23.6%), and prior authorization requirements (*n* = 16, 22.2% vs. *n* = 10, 15.2%) (Graph 2). Similar findings were observed in subgroup analyses restricted to participants who reported having at least one appointment and completed satisfaction surveys.

## Discussion

We report that decreased satisfaction with healthcare access may be associated with reduced access to medical services for children with Down syndrome during the COVID-19 pandemic. Although disruptions to medical care during the pandemic are well documented, the impact on healthcare access satisfaction has not been extensively studied [17, 22]. Reduced access to routine medical care has been associated with negative outcomes on both patients’ and caregivers’ emotional and physical well-being [23–27], as limited access heightened anxiety, feelings of isolation, and missed opportunities for timely medical interventions. Notably, 58–65% of caregivers, particularly parents of children with Down syndrome and autism, reported increased concerns about their children’s deteriorating health as a result of restricted access to healthcare services [23, 28].

The continuity of care outlined by the Health Supervision Recommendations for Children with DS was significantly disrupted during the COVID-19 pandemic [7, 8]. Pampati et al. reported that 30% of the general pediatric population had no medical care and 16% missed regular well child visits in 2020 [29]. Access to medical services declined even more among children with DS and genetic conditions. Our study found that 39.5% of the children with DS had access to a pediatrician in 2021. Likewise, genetic consultations at the Instituto Nacional de Salud del Niño in Peru decreased by 60% compared to 2019 (unpublished data, INSN-SB Galenhos System). Santoro et al. reported a reduced adherence to screenings for anemia, hypothyroidism, and ophthalmology and audiology evaluations [30].

Jeste et al. reported that 53% − 67% of patients with syndromic intellectual and developmental disabilities were able to meet at least with one provider [31], while Elmonem et al. reported a 60–80% reduction in medical appointments, including initial visits, follow-up, and therapies for patients with inborn errors of metabolism [32, 33].

In our study, our patients experienced the highest gap in medical care for cardiology, speech therapy, and early intervention, however they encountered no gaps when seeking pediatric care. Tsai et al. similarly reported reduced access to physical, occupational, and speech therapy, especially among children with DS and autism spectrum disorder (ASD) living in rural areas or from low-income families [34].

In LMICs, children with DS are particularly vulnerable to adverse health outcomes due to already limited access to healthcare [35–37]. During the pandemic, preventive and developmental services, including immunizations, therapy programs, and specialty care were deprioritized [38, 39], significantly impacting the monitoring and management of comorbidities, developmental progress, and mental health in children with DS [40, 41].

As in-person healthcare visits became inaccessible, telemedicine emerged as a viable alternative, improving healthcare access and perceived satisfaction during lockdowns [42, 43]. However, the implementation of telemedicine highlighted preexisting social inequalities such as limited internet access, lack of digital tools, and socioeconomic barriers [23]. Despite telemedicine advantages, in non-emergency situations people still prefer in person medical appointments [16]. These findings support the importance of integrating telemedicine into emergency preparedness strategies, particularly in low-resource areas [43–45].

Our study highlights pandemic-specific health care access disruptions described by other studies involving children with special healthcare needs and rare diseases. Key barriers included limited availability of specialists, delayed appointments, difficulties with referrals, prior authorizations, and transportation [46–48].

Similarly, Jeste et al. reported pharmaceutical supply chain breakdowns and insurance processing delays, with recommendations including improved access to medication, expedited specialist referrals, and home-based medication administration services [31]. Elkholi et al. documented a 94% reduction in physical therapy access, which was partially mitigated by telerehabilitation and caregiver-led therapy [49]. Aktas et al. described how hospital overcrowding and fear of infection led to postponed procedures, which were alleviated by better medication access and informal physician communication [50].

Hemmesch et al. noted additional challenges such as the need to travel for specialty care due to limited local expertise, financial constraints in accessing non-covered services, and a lack of social support during quarantine which was addressed through online support groups [51].

Given the complex medical needs of children with Down Syndrome, systematic scheduling, reliable transportation, and robust communication systems are essential. Continuity of primary care during emergencies should support appropriate referrals, specialist telemedicine coordination, and acute care delivery. Moreover, providers in general pediatrics and family medicine should receive specific training to manage care for this population, especially in emergency contexts [35–37].

This study has several limitations. First, data were collected through interviews, which may be subject to recall bias [48]. Given the disruptive impact of the COVID-19 pandemic on healthcare access, participants may have had heightened recall of events from 2021. To reduce this bias, the survey was designed with time-anchoring questions, asking about demographic and health details before satisfaction-related items. Satisfaction scores remained consistent across demographic subgroups during analysis [52, 53].

Second, selection bias was present, as 75% of the participants registered in the Association reside in the capital city. Consequently, findings may primarily reflect the experiences of urban families. As expected, these participants reported higher healthcare satisfaction than those in inner cities, especially in non-emergency contexts. Nevertheless, both urban and rural respondents reported significant declines in healthcare access and satisfaction in 2021, highlighting systemic challenges during emergencies.

Third, heterogeneity in data collection methods, via telephone or online self-administration, may have introduced variability. While this approach improved accessibility and participation, it may have contributed to incomplete responses in the online group. To mitigate this, surveys with less than 50% completion were excluded from analysis.

Fourth, survivorship bias must be considered. Families of children who died during the pandemic, and those facing greater barriers, may be underrepresented. This warrants future research focused on more vulnerable populations, including rural residents, non–Spanish-speaking families, and individuals from diverse backgrounds.

Future research should link survey data with medical records to assess alignment with clinical guidelines, providing deeper insights from LMICs. Additionally, addressing the challenges of transitioning care to adult medicine, particularly in emergency settings, is essential.

A major strength of this study is that it is the first to evaluate healthcare access and satisfaction for children with Down Syndrome during a global health emergency. These findings can inform preparedness efforts in LMICs for future crises.

## Conclusion

Healthcare access satisfaction declined during the COVID-19 public health emergency, largely due to reduced availability of medical services for children with Down Syndrome. This highlights the urgent need to identify and mitigate barriers that affect the continuity of care, including delayed appointments, difficulties with referrals, prior authorizations, medication, transportation, limited access to home medication administration and ancillary services, and communication with medical providers. Given the limited access to specialty care during crises, pediatric healthcare providers often serve as frontline responders and must be adequately trained to manage the complex comorbidities commonly associated with genetic conditions such as Down syndrome.

Telemedicine emerged as a critical tool during the pandemic and warrants further evaluation and implementation, especially in LMICs where healthcare disparities are exacerbated by geographic, communication, and transportation challenges. Strengthening telehealth infrastructure may help close persistent gaps in access to medical care for children with special healthcare needs.

Integrating targeted healthcare policies and support services will enable health systems to respond more effectively to public health emergencies while ensuring continuity of care for children with rare and genetic conditions. This study contributes to advancing awareness and supports the development of evidence-based healthcare strategies that improve access and satisfaction for children with special needs, with an emphasis on those affected by genetic disorders.

## Supplementary Information

Supplementary Material 1.

Supplementary Material 2.

Supplementary Material 3.

Supplementary Material 4.

## Data Availability

The data that supports the findings of this study is deidentified and openly available upon request.

## Abbreviations

DS: Down Syndrome
COVID-19: Coronavirus disease 2019
UN: United Nations
LMIC: Low-and-Middle income countries
AAP: American Academy of Pediatrics
IRB: Institutional Review Board
ENT: Ear, nose and throat
ASD: Autistic Spectrum Disorder
