# Supplementary material for: Healthcare access satisfaction before and during the COVID-19 pandemic among Peruvian children with down syndrome
Source: BMC Pediatr. 2025 Oct 28;25:874. doi: 10.1186/s12887-025-05990-1 (PMC12560412; doi:10.1186/s12887-025-05990-1)
Supplement: Supplementary file 1 — Supplementary Material 1. [file 12887_2025_5990_MOESM1_ESM.docx]

**APPENDIX**

**TABLE 1.** Characteristics of children with Down Syndrome and their parents.
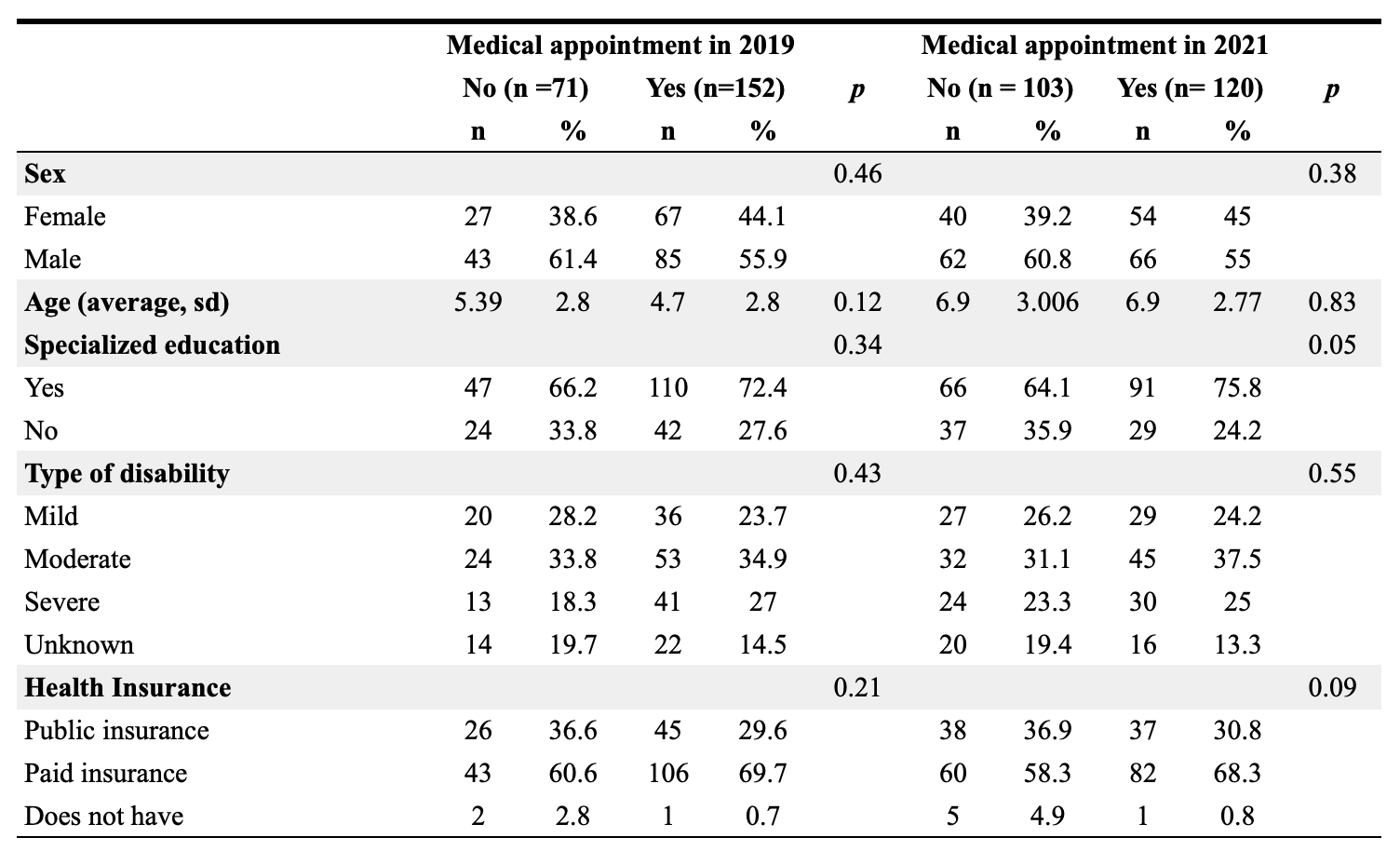


**TABLE 2.** Association between perceived satisfaction, year of medical care and demographic covariates.


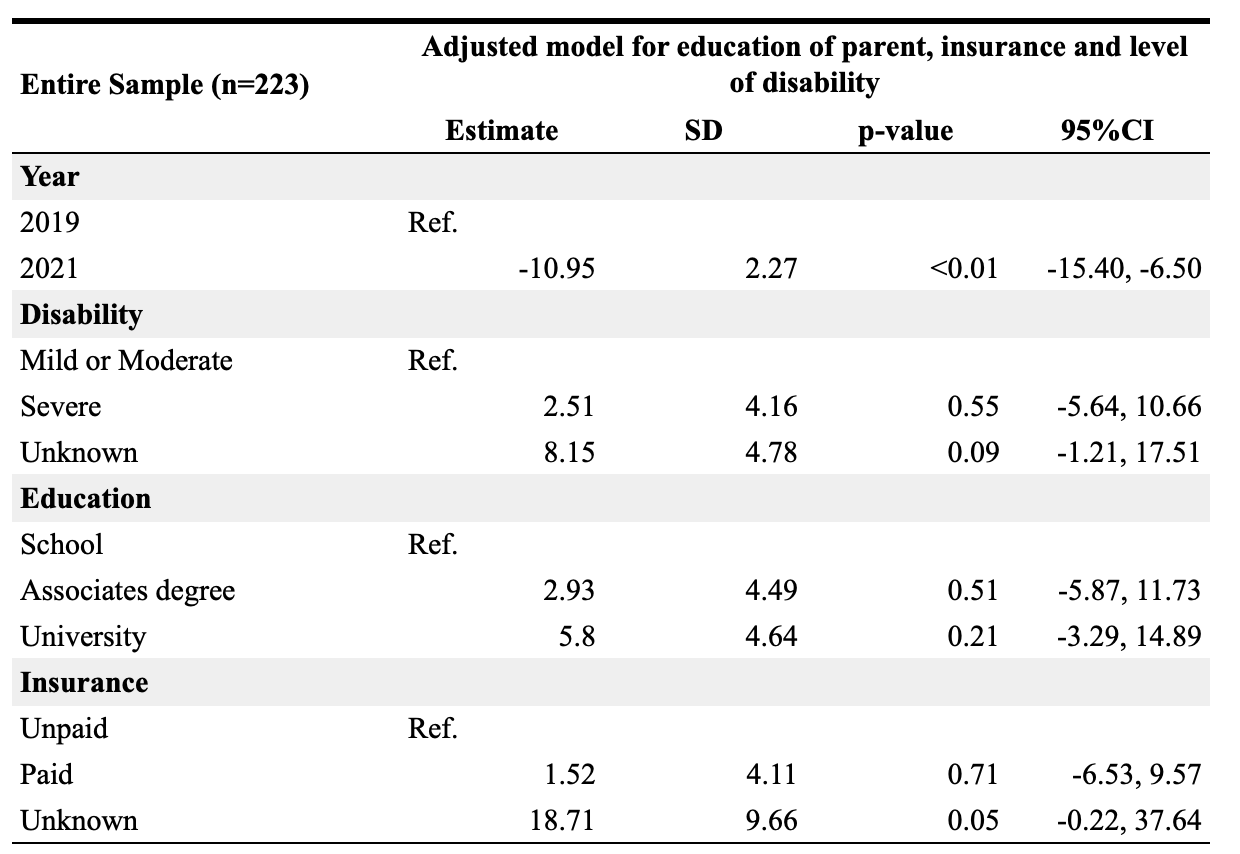


**GRAPHIC 1.** Gap in medical care for each specialty, comparing 2019 and 2021.


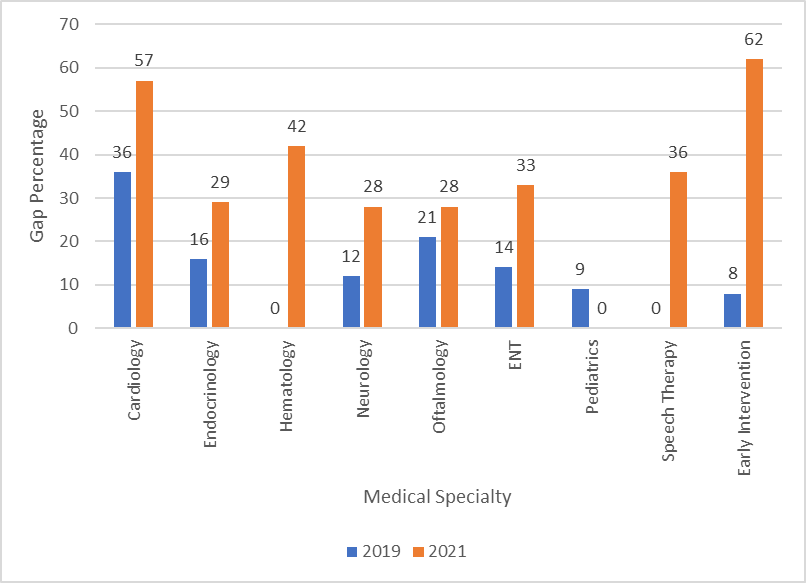


**GRAPHIC 2.** Barriers when obtaining medical care in 2019 and 2021.

**
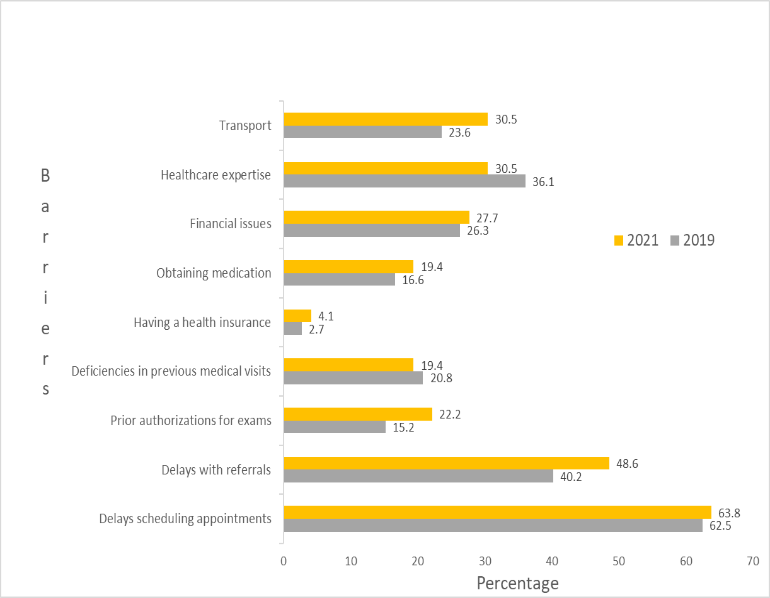
**

**SUPPLEMENTARY MATERIAL**

**TABLE A.** Characteristics of children with Down Syndrome with at least one attention in 2019 and 2021 in any specialty.


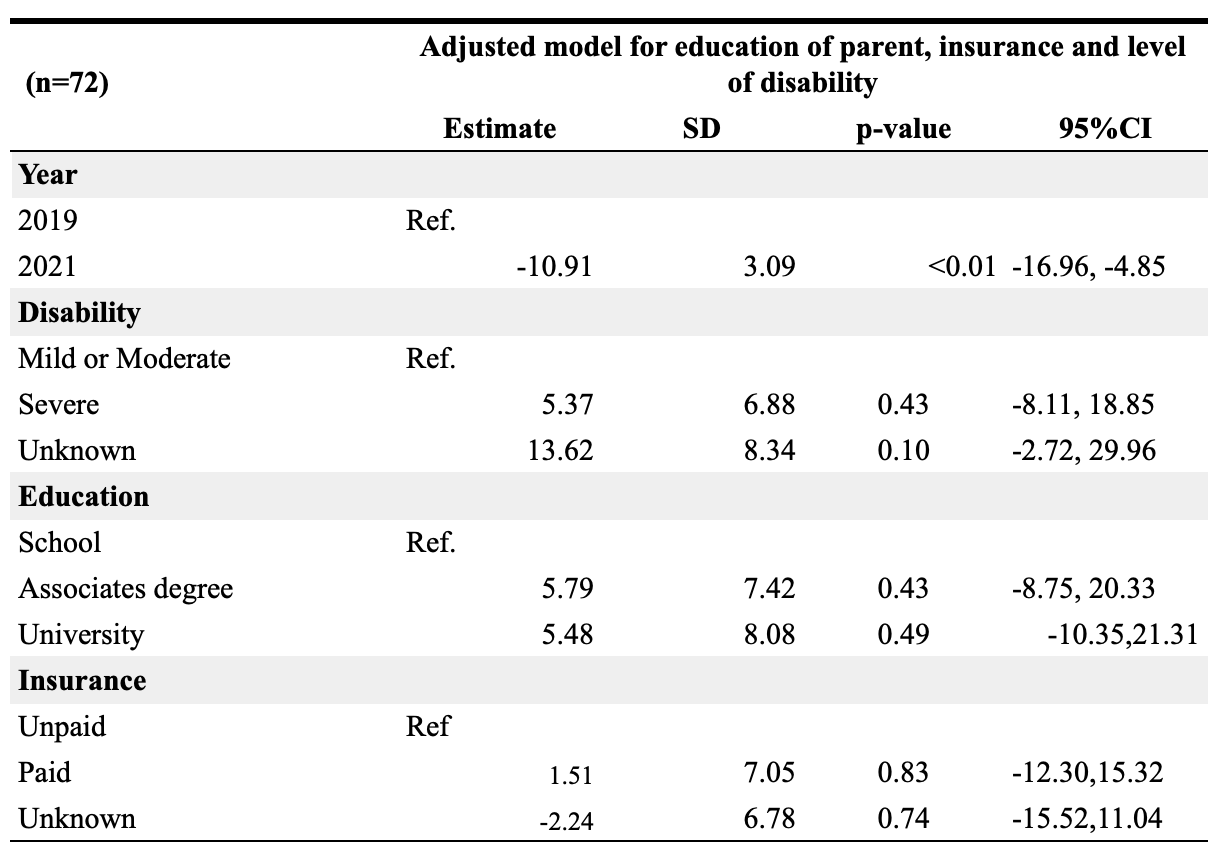


**TABLE B.** Characteristics of children with Down Syndrome with at least one attention in 2019 and 2021 in Pediatric Care.

**
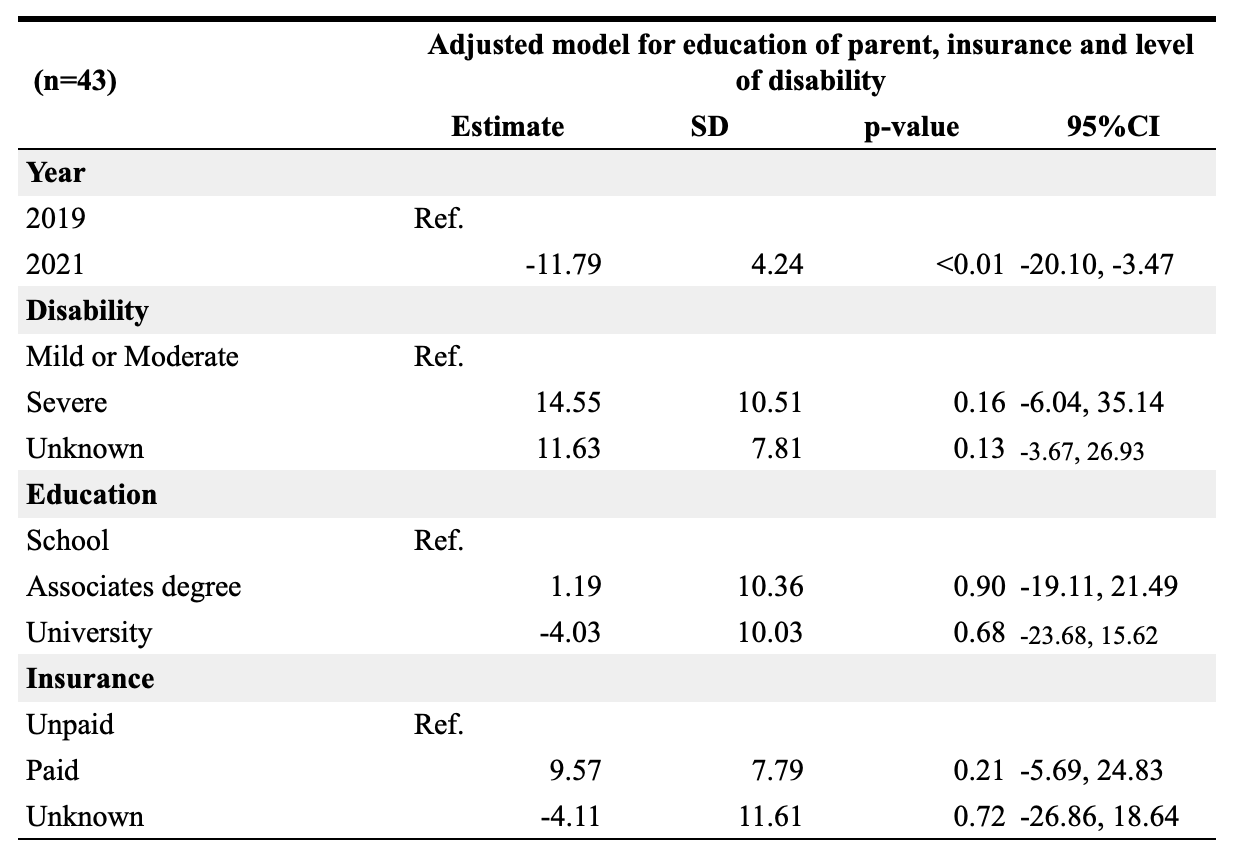
**
