# Supplementary material for: Healthcare access satisfaction before and during the COVID-19 pandemic among Peruvian children with down syndrome
Source: BMC Pediatr. 2025 Oct 28;25:874. doi: 10.1186/s12887-025-05990-1 (PMC12560412; doi:10.1186/s12887-025-05990-1)
Supplement: Supplementary file 2 — Supplementary Material 2. [file 12887_2025_5990_MOESM2_ESM.docx]

**SURVEY**

**A. Demographic Information of the child with Down Syndrome**

We would like to gather some general details about the child with Down syndrome.

1. What is the birth date of the child?
2. What is their gender?
   - Female
   - Male
3. What is their level of education?
   - Preschool
   - Completed primary school
   - Incomplete primary school
   - Completed high school
   - Incomplete high school
   - None
   - Don’t know
4. Have they ever received specialized education?
   - Yes
   - No
   - Don’t know
5. In which city was the child born?
6. Does the child have a CONADIS (National Council for the Integration of Persons with Disabilities) registration?
   - Yes
   - No
   - Don’t know

**B. Health Background of the child with Down Syndrome**

1.a. According to the CONADIS registration, what is the child’s level of intellectual disability?

- Mild
- Moderate
- Severe
- Don’t know

1.b. At their last medical visit, what was the doctor’s assessment of the child’s level of intellectual disability?

- Mild
- Moderate
- Severe
- Don’t know

1. What illnesses have they had?
   - Congenital heart disease or cardiac malformation
   - Congenital digestive malformations
   - Congenital hypothyroidism
   - Seizures or epilepsy
   - Anemia
   - Leukemia
   - Eye diseases
   - Delayed language development
   - Delayed psychomotor development
   - Oral cavity disorders
   - Hearing problems
   - Other: ___________
2. If you selected "Other," please specify: ___________

If "Congenital heart disease" was selected, did the doctor recommend surgery?

- Yes
- No
- Don’t know

Did they have heart surgery?

- Yes
- No, still pending
- No, not needed
- Don’t know

**C. Access to Healthcare Services Before the Pandemic (2019)**

We will now ask about the child’s general health and healthcare access in 2019 before the pandemic.

1. Where did the child live in 2019?
   - At home with family
   - In a shelter
   - Other: ___________
2. In which city did they live in 2019?
3. What type of health insurance did they have in 2019?
   - Public Health Insurance (SIS)
   - Social Health Insurance (ESSALUD)
   - Military Insurance
   - Private Insurance
   - None
   - Don’t know
4. In 2019, did the child experience any health concerns that required a doctor’s visit?
   - Yes
   - No
   - Don’t know
5. If yes, which medical specialties were needed?
   - Cardiology
   - Surgery
   - Endocrinology
   - Gastroenterology
   - Medical Genetics
   - Hematology
   - General Medicine
   - Neurology
   - Ophthalmology
   - Otolaryngology
   - Pediatrics
   - Speech Therapy
   - Early Intervention Therapy
   - Other: ___________
6. From the above specialties, which ones provided medical attention?
   - Cardiology
   - Surgery
   - Endocrinology
   - Gastroenterology
   - Medical Genetics
   - Hematology
   - General Medicine
   - Neurology
   - Ophthalmology
   - Otolaryngology
   - Pediatrics
   - Speech Therapy
   - Early Intervention Therapy
   - Other: ___________
7. How were these medical appointments conducted? (select all that apply)
   - In-person at a clinic
   - By phone or video call
   - Did not receive medical attention
   - Don’t know
8. In 2019, was the child diagnosed with anemia or low hemoglobin?

- Yes
- No
- Don’t know

1. If yes, did they receive treatment for anemia?

- Yes
- No
- Don’t know

1. In 2019, did the child have thyroid problems?

- Yes
- No
- Don’t know

1. If yes, did they receive treatment for thyroid problems?

- Yes
- No
- Don’t know

1. What barriers made accessing healthcare difficult in 2019?

- Long wait times for appointments
- Issues with referral systems
- Difficulties obtaining test authorizations
- Insurance enrollment difficulties
- Poor quality of past medical consultations
- Difficulty obtaining medications
- Financial problems
- Healthcare staff lacked knowledge about Down syndrome
- Difficulty transporting the child to the healthcare facility

1. In 2019, how satisfied were you with the medical care received? (Scale of 1-5, where 1 is the worst and 5 is the best)

**D. Access to Healthcare Services During the Pandemic (2021)**

We will now ask about the child’s healthcare access in 2021, during the COVID-19 pandemic.

1. Did the child’s place of residence change due to COVID-19?
   - Lived at home with family
   - In a shelter
   - Other: ___________
2. In which city did they live in 2021?
3. What type of health insurance did they have in 2021?
   - Public Health Insurance (SIS)
   - Social Health Insurance (ESSALUD)
   - Military Insurance
   - Private Insurance
   - None
   - Don’t know
4. In 2021, did the child experience any health concerns that required a doctor’s visit?
   - Yes
   - No
   - Don’t know
5. If yes, which medical specialties were needed?
   - Cardiology
   - Surgery
   - Endocrinology
   - Gastroenterology
   - Medical Genetics
   - Hematology
   - General Medicine
   - Neurology
   - Ophthalmology
   - Otolaryngology
   - Pediatrics
   - Speech Therapy
   - Early Intervention Therapy
   - Other: ___________
6. From the above specialties, which ones provided medical attention?
   - Cardiology
   - Surgery
   - Endocrinology
   - Gastroenterology
   - Medical Genetics
   - Hematology
   - General Medicine
   - Neurology
   - Ophthalmology
   - Otolaryngology
   - Pediatrics
   - Speech Therapy
   - Early Intervention Therapy
   - Other: ___________
7. How were these medical appointments conducted?
   - In-person at a clinic
   - By phone or video call
   - Did not receive medical attention
   - Don’t know
8. In 2021, was the child diagnosed with anemia or low hemoglobin?

- Yes (Please, go to next question)
- No (Please, go to question 10)
- Don’t know

1. If yes, did they receive treatment for anemia?

- Yes
- No
- Don’t know

1. In 2021, did the child have thyroid problems?

- Yes (Please, go to question 11)
- No (Please, go to question 12)
- Don’t know

1. If yes, did they receive treatment for thyroid problems?

- Yes
- No
- Don’t know

1. What barriers made accessing healthcare difficult in 2021?

- Long wait times for appointments
- Issues with referral systems
- Difficulties obtaining test authorizations
- Insurance enrollment difficulties
- Poor quality of past medical consultations
- Difficulty obtaining medications
- Financial problems
- Healthcare staff lacked knowledge about Down syndrome
- Difficulty transporting the child to the healthcare facility

1. How satisfied were you with the medical care received in 2021? (Scale of 1-5)

**E. Information About the Primary Caregiver**

1. What is your relationship to the child?

- Mother
- Father
- Sibling
- Uncle/Aunt
- Grandparent
- Cousin
- Other: ___________

1. Are you the primary caregiver?

- Yes
- No

1. What is your age?
2. What is your gender?

- Feminine
- Masculine
- Other: _________

1. What is your level of education?

- Preschool
- Primary school
- Secondary school
- Technical studies
- University
- None
- Don’t know

1. Where do you get information about medical care for a person with Down syndrome?

- Healthcare provider
- Internet
- Groups of people with family members who have Down syndrome
- Other: ___________

Thank you for participating in this survey!
