# Supplementary figures and images for: Healthcare access satisfaction before and during the COVID-19 pandemic among Peruvian children with down syndrome
Source: BMC Pediatr. 2025 Oct 28;25:874. doi: 10.1186/s12887-025-05990-1 (PMC12560412; doi:10.1186/s12887-025-05990-1)

Supplementary Figure: Selection of study sample

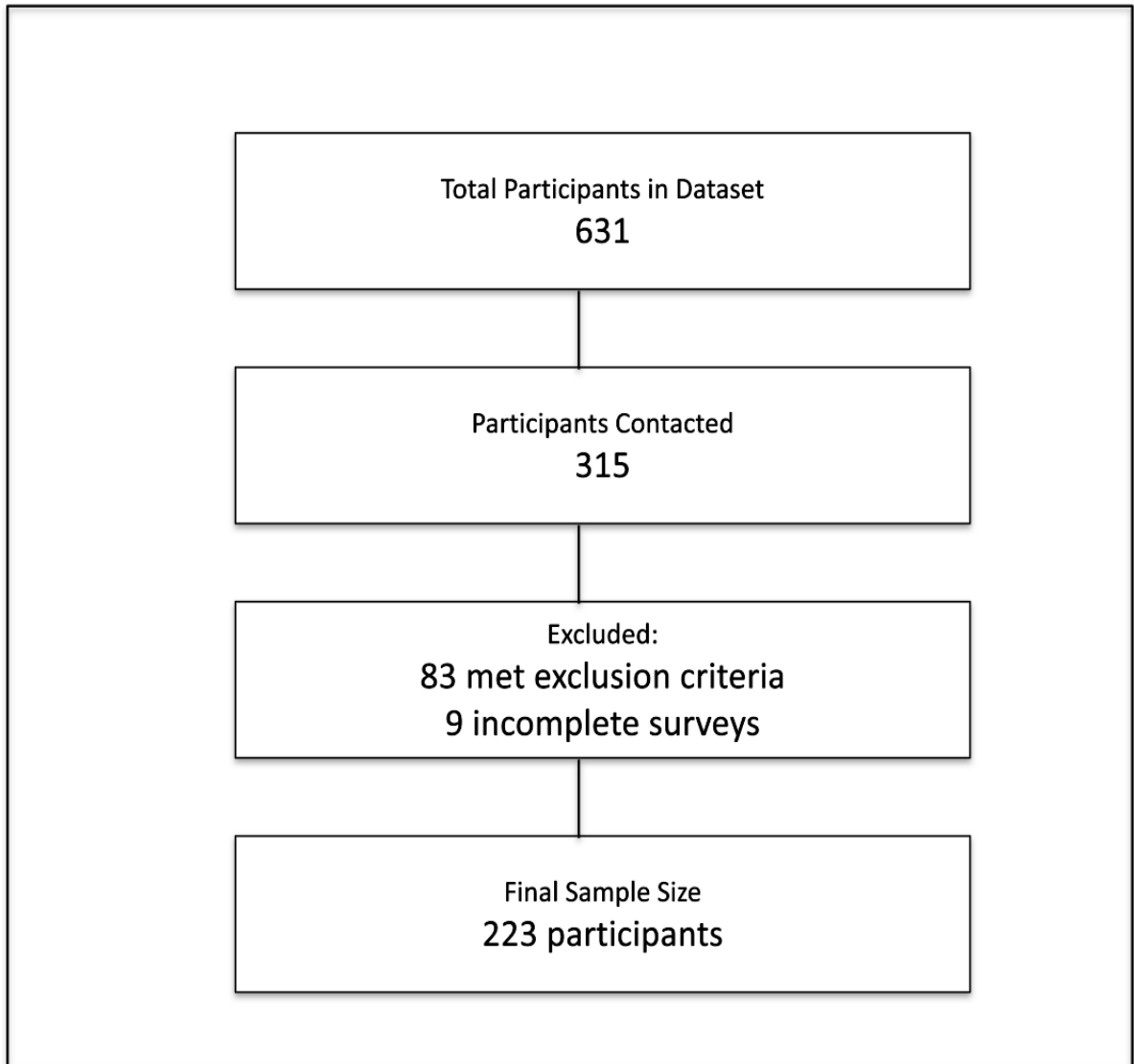

Supplement: Supplementary file 4 — Supplementary Material 4. [file 12887_2025_5990_MOESM4_ESM.pdf]
